# Late Post-LASIK visual Deterioration

**Published:** 2010-10

**Authors:** Farid Karimian, Amir Faramarzi, Afshin Lotfi

**Affiliations:** Ophthalmic Research Center, Shahid Beheshti University of Medical Sciences, Tehran, Iran; Ophthalmic Research Center, Shahid Beheshti University of Medical Sciences, Tehran, Iran; Tabriz University of Medical Sciences, Tabriz, Iran

## CASE PRESENTATION

A 40 year-old woman was referred to a refractive surgery clinic complaining of blurred vision and photophobia. She had undergone myopic LASIK 8 years ago. There had been no problem one year after surgery with best corrected visual acuity (BCVA) of 20/20 at that time. Her complaints started gradually and progressed thereafter. She had no history of systemic disease including diabetes mellitus and hypertension.

Upon presentation, BCVA in her right and left eyes were 4/10 and 5/10, with −5.00–1.50×180° and −5.50–1.50×165°, respectively. On slitlamp examination, the LASIK flap interface was barely visible. Intraocular pressure (IOP) was 14 and 13 mmHg, in the right and left eyes respectively. Funduscopy revealed mild myopic chorioretinal degeneration but was otherwise unremarkable.

What are the differential diagnoses and what would you suggest for further evaluation?

### Farid Karimian, MD

Differential diagnoses include the following:

1. Post-LASIK progressive keratectasia which most often occurs 6 to 24 months after LASIK. Good BCVA of 20/20 for one year and then its gradual decrease makes this diagnosis probable. At this stage, we are unaware of the preoperative refraction, pachymetry, and topography; thus we have little information about the risk factors for keratectasia. BCVA of 4/10 and 5/10 are in favor of keratectasia. On the other hand, regular with-the-rule astigmatism and the normal slitlamp examination are against such a diagnosis. We need a new topography to evaluate ectasia.
2. Cataract formation is another possibility. Cataracts are more common in high myopic patients and are probably seen earlier than normal age-matched persons. The progressive myopia may be due to gradual nuclear sclerosis.
3. Considering the presence of high myopia before refractive surgery, the risk of primary open-angle glaucoma is increased. IOPs are underestimated after keratorefractive procedures for myopia. To compensate for erroneous IOP measurements, 1 mmHg should be added to measured IOPs for each 14 to 17 microns of laser ablation. Alternatively, for each diopter of myopic correction, 1 to 1.5 mmHg may be added. In eyes with previous myopic refractive surgery, IOP measurement from the corneal periphery may be more accurate. The dynamic contour (Pascal) tonometer may also be more precise in such cases. The apparently normal appearance of the optic nerve head is against a diagnosis of glaucoma.
4. Long-term regression is possible in eyes that have undergone LASIK surgery for high myopia. Predisposing factors include the use of certain types of excimer machines, ablation pattern (broad beam excimers have the highest, while flying spot machines have the lowest risk of regression), optical and transition zone diameters (the lower the diameter, the higher the risk), and patient age (the younger the patient, the higher the risk). In this case with gradual regression over several years, we do not know the initial refraction or the type of the excimer machine. BCVA was 20/20 after surgery, but is not better than 5/10 now. Therefore, the probability of pure regression is low.
5. Flap wrinkling and epithelial ingrowth can also decrease visual acuity. Usually, decreased vision due to such factors occurs sooner. Both conditions can be identified on slitlamp examination, which has been normal in this case.

### Amir Faramarzi, MD

Based on history and symptoms, the most probable diagnoses are post-LASIK keratectasia, nuclear sclerosis, and post-LASIK regression. If the lens is clear on slitlamp biomicroscopy, I would request the patient’s preoperative documents, a new corneal topography, and ultrasonic pachymetry for both eyes.

### Afshin Lotfi, MD

Due to good visual acuity in the first postoperative year, gradual decrease in BCVA and gradual regression of myopia and astigmatism, the first and most important differential diagnosis is ectasia. Given her complaints, other diagnoses such as decentered ablation, regression, and corneal irregularity should also be considered. Performing a normalized topography (by changing the scales and color bars) and comparing it with preoperative topographic maps (i.e. difference map) is important. It would also be helpful to request elevation topography.

## DIAGNOSIS

**Figure 1:**
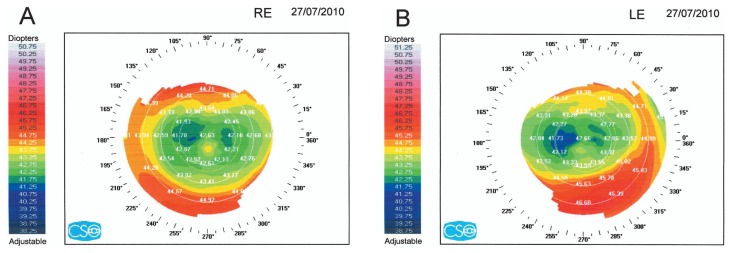
Current corneal topographic maps of the right (A) and left (B) eye.

**Figure 2:**
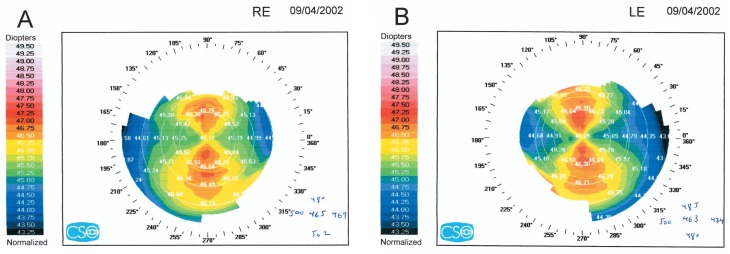
Pre-LASIK corneal topographic maps of the right (A) and left (B) eye.

**Figure 3:**
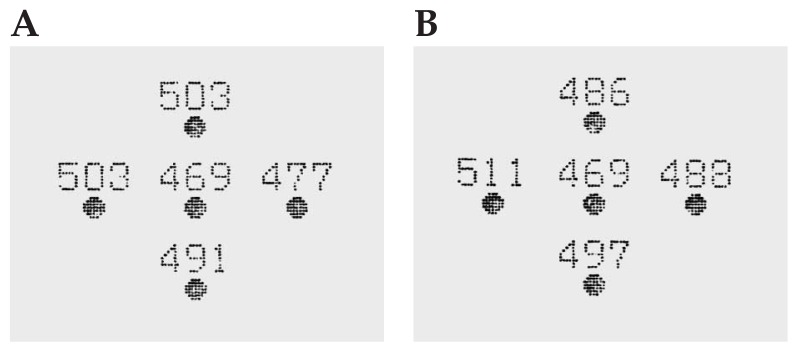
Preoperative pachymetry in the right (A) and left (B) eye.

**Figure 4:**
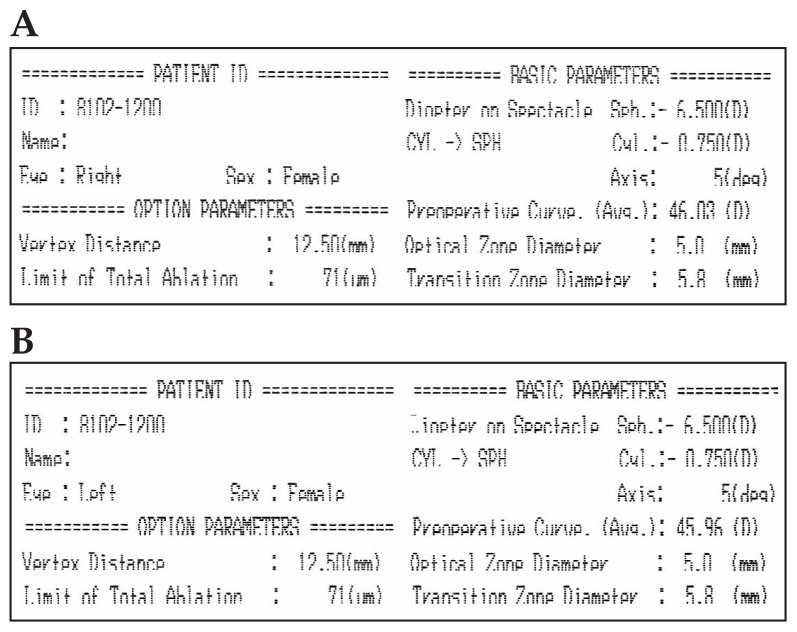
Basic surgical parameters in the right (A) and left (B) eye.

Conventional topography was requested (Figures 1A and 1B) and the patient’s records were obtained. Pre-LASIK subjective refraction had been −8.50–1.50×5° and −8.75–2.00×5°, in the right and left eyes respectively, and BCVA had been 20/20 in both eyes. Full cycloplegic refraction had been −8.00–1.50×5° and −8.00–1.75×10° in her right and left eyes respectively. Preoperative corneal topography (Figures 2A and 2B), and pachymetry (Figures 3A and 3B) are shown. LASIK had been performed using the Nidek EC-5000 (Nidek Co. Ltd., Gamagori, Japan) excimer laser machine. Basic surgical parameters are shown in figures 4A and 4B.

What is your final diagnosis and how would you explain her problems?

### Farid Karimian, MD

In the excimer laser printout, considering a flap thickness of 140 microns, target refraction for surgery has been reduced to keep at least 250 microns of residual stroma; in both eyes −6.5 D of myopia and −0.75 D of astigmatism were the attempted correction. Therefore, 2.5 D of myopia and 0.75 to 1.00 D of astigmatism should have remained. There are two problems with this plan. First is the small optical and transition zones employed (5.0 and 5.8 mm, respectively) which may predispose to myopic regression. Second is the use of a scanning slit excimer laser system. The risk of myopic and astigmatic regression is higher with scanning slit machines as compared to their flying spot counterparts.

Recent topographic maps demonstrate a hot ring in the periphery of the cornea suggesting regression. Serial topographic maps would have better been able to demonstrate this issue. Of importance are the non-homogeneous topographic areas in the central cornea near the visual axis. In other words, there is irregular astigmatism which can explain limited BCVA. Therefore, my final diagnosis is not simply myopic regression; other factors including a small optical zone and irregular astigmatism have further aggravated the condition.

### Amir Faramarzi, MD

Preoperative topography shows symmetric bow-tie pattern in both eyes. The power of the steepest meridian is less than 47 diopters. Recent topography shows central corneal flattening, but with small central islands in both eyes. According to these topographies, post-LASIK keratectasia can be ruled out. The most probable diagnosis is central island in both eyes.

Because of the high refractive error and thin cornea, target ablation had been reduced by approximately 2 D. The optical zone was also decreased to 5.0 mm to reduce the amount of ablation. Small optical zones can cause glare, poor contrast sensitivity and unsatisfactory visual performance. In addition the risk of regression is increased. Although central islands occur more commonly with older broad beam laser delivery systems, I think the main cause of the patient’s symptoms is central corneal island which has led to decreased BCVA.

Other causes such as undercorrection and regression must also be considered. Distinguishing iatrogenic keratectasia from a steep central island is very important since retreatment of keratectasia may exacerbate the ectatic process. In suspicious cases especially in the early postoperative period, it would be logical to wait at least 6 months, because central islands usually improve over time (90% of topographic islands following photorefractive keratectomy resolve by 6 months). However, post-LASIK ectasia aggravates over time. Aberrometry must be performed, which will most probably show significant higher order aberrations.

### Afshin Lotfi, MD

The optical zones are small in both eyes. Due to central corneal thinning, therapeutic protocols had limitations and the refractive error was not fully corrected. This could have caused residual refractive error and corneal irregularity. Wavefront (especially corneal) analysis will help identify regression and corneal irregularity.

Was there any better surgical alternative at the time of LASIK?

### Farid Karimian, MD

With the Nidek EC-5000 excimer laser machine, a multi-zone strategy would have been beneficial. Photorefractive keratectomy (PRK) enhanced by mitomycin C (MMC) would have also been safer than LASIK.

Based on current knowledge, anterior chamber (AC) depth measurement using Orbscan (Bausch & Lomb, Orbtek Inc., Salt Lake City, UT, USA) or one of the Scheimpflug cameras, i.e. Pentacam (Oculus, Wetzlar, Germany) or Gallilei (Ziemer, Port, Switzerland), would have been necessary. In case of adequate AC depth (more than 3 mm), and adequate endothelial cell count (more than 2,500), phakic intraocular lens implantation could have been a viable alternative. In the presence of astigmatism, a Toric implantable collamer lens (Toric ICL, STAAR, Monrovia, CA, USA) or iris fixation intraocular lenses (IOLs) such as Artisan or Artiflex (Ophtec BV, Groningen, The Netherlands) were other options. Due to low corneal thickness, bioptics (excimer laser surgery after phakic IOL implantation) is not recommended. For ICL implantation, sulcus-to-sulcus measurement using ultrasound biomicroscopy (UBM) and corneal white-to-white diameter measurement using calipers or the Orbscan/Pentacam is recommended to prevent complications.

### Amir Faramarzi, MD

At that time, one could have performed PRK with MMC augmentation; however, in my opinion, the best choice for high myopia (more than 8 D) is phakic IOL implantation. Artisan phakic IOL was available at that time and if AC depth had been adequate, I would have chosen Artisan phakic IOL implantation.

### Afshin Lotfi, MD

Considering the excimer machine employed, a multi-zone protocol could have helped her attain a regular multifocal cornea. On the other hand, due to corneal thinning, phakic IOLs may have been a better choice. However, clear lens extraction is not recommended at this age.

What line of treatment would you suggest now?

### Farid Karimian, MD

If there is no ocular or systemic contraindication, rigid gas-permeable lenses (RPGs) are helpful for correcting irregular astigmatism. Despite claims by the manufacturers, different surface ablations even of the wavefront-guided type are not very successful, especially in this case, due to high residual refractive error and corneal thinning. Due to the origin of the irregular astigmatism which is in the cornea, IOLs would not be helpful. They may decrease the amount of myopia, but will not improve BCVA. Soft contact lenses will also be ineffective.

There are problems with RGP contact lens fitting in patients with previous LASIK. Many such patients decide to perform refractive surgery due to contact lens intolerance or their complications. Hence, returning to RGP lenses is troublesome. On the other hand, due to over-flattening of the central cornea and steepening in the mid-periphery, RGP fitting is difficult. Air-bubbles form behind the lens or the edge of the contact lens is slabbed off, and the lens may fall away from the eye. In case of intolerance to contact lenses, corneal transplant surgeries, including penetrating or lamellar keratoplasty, should be considered which have their own limitations.

### Amir Faramarzi, MD

Contact lenses, especially RGPs, may improve the symptoms and are best tried first. However, if the patient cannot tolerate them or demands surgery, I would recommend wavefront-guided or topography-guided surface ablation with mitomycin application. If current corneal thickness (including the epithelium) is at least 400 microns, it is safe to perform advanced wavefront-guided surface ablation over the LASIK flap.

Wavefront guided ablation can decrease the refractive error, reshape the cornea into a smoother surface, and improve higher order aberrations. However, further stromal ablation theoretically increases the risk of iatrogenic keratectasia. Due to unreliability of objective or subjective refraction in this case, the final refractive state is unpredictable.

### Afshin Lotfi, MD

The approach to patients with corneal surface irregularity (decentration, small optical zone, etc.) depends on their symptoms (photophobia, halo vision, night vision problems, studying problems, etc.), amount of loss of BCVA, changes in quality of life, and aberrometric indices. In case of mild irregularity, observation is recommended, but with to 3 to 4 lines decrease in BCVA, I would prefer to perform corneal wave-front analysis. This could quantify the amount of corneal surface irregularity which can be corrected, without correcting the remaining or regressed refractive error. This procedure may improve BCVA which is dependent on the amount of P-V in corneal wavefront analysis. Refractive error should be adjusted according to surgeon’s nomogram (if P-V value is between 10 to 20 microns, +2 to +3.00 hyperoptic shift is induced during correction of the corneal irregularity). Therefore, residual refraction after correction of corneal surface irregularity should be between −2.0 to −3.0 D. This may require glasses, contact lenses, or separate/simultaneous refractive surgery on the corneal or lens plane. The limiting factor in this case is corneal thickness. Lens surgery without correcting corneal surface irregularity will not change BCVA.

